# A New Isorhamnetin Glycoside and Other Phenolic Compounds from *Callianthemum taipaicum*

**DOI:** 10.3390/molecules17044595

**Published:** 2012-04-17

**Authors:** Dong-Mei Wang, Wen-Jun Pu, Yong-Hong Wang, Yu-Juan Zhang, Shan-Shan Wang

**Affiliations:** 1 College of Forestry, Northwest A & F University, Yangling 712100, China; Email: Yvonne2nadal@163.com (W.-J.P.); hedwig.yujuan@gmail.com (Y.-J.Z.); 121268525@qq.com (S.-S.W.); 2 Shanghai Chempartner Co., LTD, Shanghai 201203, China; Email: Yhwang@chempartner.cn

**Keywords:** *Callianthemum taipaicum*, isorhamnetin glycoside, phenolic compounds, antifungal activity

## Abstract

A new flavonol glycoside together with five known phenolic compounds were isolated from the whole herb of *Callianthemum taipaicum*.The compounds were identified as isorhamnetin-3-*O*-α-L-arabinoside-7-*O*-β-D-glucoside (**1**), isorhamnetin-3-*O*-β-D-glucoside (**2**), dibutyl phthalate (**3**), (+)-1-hydroxylpinoresinol-4'-β-D-glucoside (**4**), pinoresinol-4'-*O*-β-D-glucoside (**5**) and 2-phenylethyl-β-primeveroside (**6**). Compound **1** was identified as a new flavonol glycoside. The compound **6** was isolated for the first time as natural product. All compounds were isolated for the first time from the *Callianthemum* genus. Furthermore, the 2D-NMR data of the four known compounds **2–5** are given for the first time in this paper. All the structures were identified on the basis of detailed spectral analysis. The compounds **1** and **4** exhibited certain antifungal activity.

## 1. Introduction

Plants have been utilized as medicines for thousands of years [1]. About 80% of the World’s population uses medicinal plants for primary health care [2]. Medicinal plants have attracted more and more attention for their “safety, cultural acceptability and lesser side effects”, for instance, *Ginkgo biloba* for tinnitus, *Hypericum perforatum* for depression [3]. Natural products isolated from medicinal plants were used by indigenous populations for physiological and therapeutic effect, aconitine, atisine, lstrychnine, digoxin, atropine and morphine are some common examples [4]. Isolation and characterization of natural products from medicinal plants has played a significant role in drug discovery and development.

The Qinling Mountains are an important traditional Chinese medicine area in China. They serve as an important medicinal plant resource. Taibai Mountain, also known as “Herbal Kingdom” and “Natural Botanical Garden of Asia”, is the highest peak of the Qinling Mountains, with the altitude of 3767.2 m. Due to the special environment, there are many unique medical plants growing in this area [5]. The *Callianthemum* genus belongs to the family Ranunculaceae, which include 12 species worldwide, many of them are found in Asia and Europe. There are five species distributed in west and northwest China. *Callianthemum taipaicum* is an endemic species of Taibai Mountain, where it grows at altitudes between 3,450 to 3,600 m [6]. The whole herb of this plant has detoxifying and anti-inflammatory activity, and can be used as a medicine to treat various diseases including children’s pneumonia and drug-fire eyesight [7]. Due to the special environment where *Callianthemum taipaicum* grows, it could contain many special phytochemicals. However, a literature survey revealed that no chemical work has been carried out on this plant or indeed the *Callianthemum* genus so far. In order to identify functional compounds from *Callianthemum taipaicum*, we have isolated a new isorhamnetin glycoside (**1**) together with five known phenolic compounds **2–6**. The present paper, for the first time, describes the isolation and structure elucidation of these compounds by detailed analysis of their NMR spectra. The antifungal activity of compounds **1**, **4** is also discussed.

## 2. Results and Discussion

### 2.1.Structure Elucidation of the New Compound

**Figure 1:**
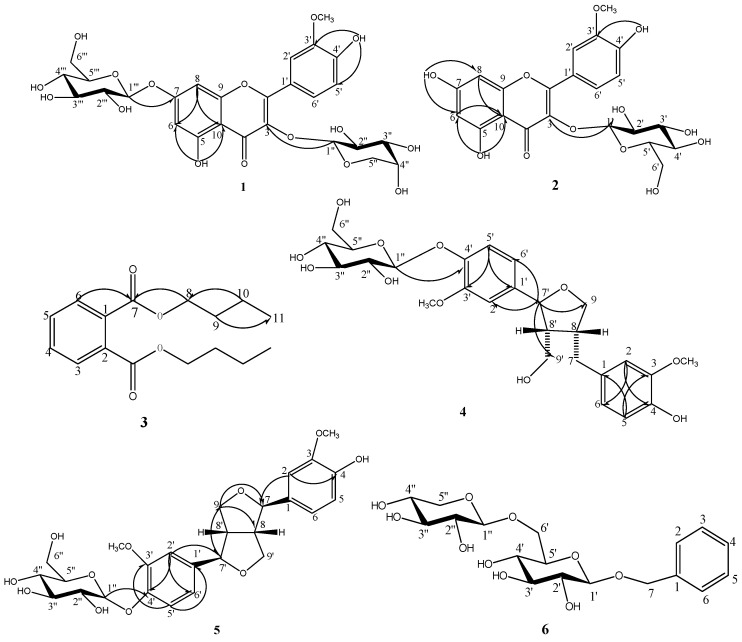
The structure and the key HMBC correlations of compounds **1–6**.

Compound **1** obtained as a light yellow amorphous powder. Its molecular formula was established as C_27_H_30_O_16_ on the basis of HR-ESI-MS: *m/z* 611.1602 [M+H]^+^ (calcd. [M+H]^+^ 611.1607) and ESI-MS *m/z* 611.0 [M+H]^+^. The IR spectrum showed the characteristic absorption bands of hydroxyl (3444.87 and 3429.43 cm^−1^), carbonyl (1653.00 cm^−1^) and phenyl group (1600.92 and 1490.97 cm^−1^) [8]. ^13^C-NMR and DEPT spectrum showed signals for one OCH_3_, two CH_2_ (all aliphatic), fourteen CH (five aromatic, nine aliphatic) and ten quaternary carbons (one carbonyl, five O-bearing and four aliphatic) [9]. The ^1^H- and ^13^C-NMR spectra of compound **1** were compared with the data of known compounds and showed a typical flavonol pattern with an isorhamnetin aglycon (Table 1) [10,11,12]. In addition to the isorhamnetin moiety, mass and NMR spectra showed signals corresponding to two sugar moieties. Acid hydrolysis of compound **1** gave D-glucose and L-arabinose, identified by comparision with an authentic sample. The sugar portion was examined by TLC analysis. The ^1^H-NMR spectrum of compound **1** displayed two doublets at δ 5.57 (1H, d, *J* = 7.0 Hz) and δ 5.06 (1H, d, *J* = 6.6 Hz) for the anomeric protons. Based on the coupling constant *J* = 7.0 Hz and 6.6Hz greater than 4.0 Hz, the sugar configurations could be identified as α-L-arabinose and β-D-glucose, respectively, which correlated respectively with signals at 100.71, 100.11 in the HSQC spectrum [13,14]. In the HMBC (Figure 1), the anomeric proton signal of the arabinose at δ 5.57 (H-1'') correlated with the carbon signal at δ 133.26 (C-3), the proton signal of the glucose at δ 5.06 (H-1''') correlated with the anomeric carbon signal of the apiose at δ 162.73(C-7). These indicated that the arabinose attached to C-3 of the aglycone, the glucose attached to C-7 of the aglycone [15]. In addition, in the literature, there was a remarkable upfield shift (∆δ 2.7) for the C-7 of an isorhamnetin 3,7-diglycoside (δ 161.8) compared to an isorhamnetin 3-glycoside (δ 164.5), the ^13^C-NMR spectrum of compound **1** showed that the chemical shift of δ 162.73 for C-7, was close to the chemical shift of δ 161.8 [11]. Based on the above evidence and detailed analysis of the NMR spectra, the structure of compound **1** was determined as isorhamnetin-3-*O*-α-L-arabinopyranose-7-β-D-glucopyranoside, which had not been reported previously.

**Table 1:** ^1^H-, ^13^C-NMR and HMBC spectral data of compound 1 (in DMSO, *δ* in ppm, *J* in Hz).

Structure	Position	Compound 1			Isorhamnetin 3- *O*-β-D-glucoside-7-*O*-α-L-rhamnoside	
		δ _H_	δ _C_	HMBC	δ _H_	δ _C_
Flavonol group	2		156.79			156.9
	3		133.26			133.3
	4		177.56			177.6
	5	12.6 (1H, s)	160.85	C-5,6,10		161.0
	6	6.43 (1H, d, 1.5 Hz)	99.27	C-5,7,8,10	6.44 (1H, d, 2.0 Hz)	99.4
	7		162.73			161.6
	8	6.97 (1H, d, 1.5 Hz)	94.49	C-6,7,9,10	6.83(1H,d, 2.0 Hz)	94.7
	9		155.97			156.0
	10		105.59			105.7
	1'		120.92			121.0
	2'	7.97 (1H, s)	113.54	C-2,3',6'	7.94 (1H, s)	113.5
	3'		149.58			149.7
	4'	9.87 (1H, s)	146.90	C-3',5'		147.0
	5'	6.93 (1H, d, 8.5 Hz)	115.20	C-1',3'	6.93 (1H, d, 8.5 Hz)	115.3
	6'	7.54 (1H, d, 8.5 Hz)	122.19	C-2,2',3'	7.55 (1H, d, 8.5 Hz)	122.4
	3'-OCH_3_	3.84 (3H, s)	55.69	C-3'	3.83 (3H, s)	55.8
Sugar at C-3	1''	5.57 (1H, d, 7.0 Hz)	100.71	C-3	5.57 (1H, d, 6.7 Hz)	100.8
	2''	3.0–3.8 (1H, m)	74.32		3.2 (1H, m)	74.4
	3''	3.0–3.8 (1H, m)	67.32		3.2 (1H, m)	76.5
	4''	3.0–3.8 (1H, m)	69.81		3.1 (1H, m)	70.1
	5''	3.0–3.8 (2H, m)	60.60		3.1 (1H, m)	77.6
	6''	-------	-----	-----	3.57, 3.45	60.6
Sugar at C-7	1'''	5.06 (1H, d, 6.6 Hz)	100.11	C-7	5.55 (1H, s)	98.4
	2'''	3.0–3.8 (1H, m)	70.00		3.83 (1H, s)	70.1
	3'''	3.0–3.8 (1H, m)	76.40		3.63 (1H, d, 3.2 Hz)	70.3
	4'''	3.0–3.8 (1H, m)	72.33		3.3 (1H, m)	71.7
	5'''	3.0–3.8 (1H, m)	77.45		3.4 (1H, m)	69.9
	6'''	3.0–3.8 (2H, m)	65.72		1.10 (1H, d, 6.1 Hz)	18.0

### 2.2. Antifungal Activity of Compounds

Due to the insufficient quantities of compounds **2**, **3**, **5**, **6**, we only tested compounds **1** and **4** for their antifungal activities using amphotericin as positive control (Table 2). The results showed that the tested compounds exhibited certain antifungal activity. Compound **1** showed inhibitory activity against the growth of *Rhizoctonia cerealis*, *Botrytis cinerrea and Thanatephorus cucumeris* with EC_50_ was 1.92 ± 0.30, 1.38 ± 0.26, 1.39 ± 0.19 mg/mL, respectively. Compound **4** showed obviously inhibitory activity against the growth of *Rhizoctonia cerealis*, *Botrytis cinerrea and Valsa mali* with EC_50_ was 0.75 ± 0.37, 0.82 ± 0.05, 1.28 ± 0.34 mg/mL, respectively. 

**Table 2:** Antifungal results of compounds **1** and **4**.

Sample	Species	Toxicity egressions	r	EC_50_ mg/mL
Compound **1**	*Rhizoctonia cerealis*	y = 16.004x + 19.444	0.9945	1.92 ± 0.30
	*Botrytis cinerrea*	y = 31.509x + 6.5604	0.9322	1.38 ± 0.26
	*Thanatephorus cucumeris*	y = 31.145x + 6.793	0.9366	1.39 ± 0.19
Compound **4**	*Rhizoctonia cerealis*	y = 62.96x + 10.36	0.9958	0.75 ± 0.37
	*Botrytis cinerrea*	y = 48.363x + 10.255	0.9768	0.82 ± 0.05
	*Valsa mali*	y = 21.612x + 23.711	0.9819	1.28 ± 0.34
Amphotericin	*Rhizoctonia cerealis*	y = 637.44x − 6.3644	0.8097	0.13 ± 0.003
	*Botrytis cinerrea*	y = 575.85x − 17.957	0.9008	0.17 ± 0.041
	*Thanatephorus cucumeris*	y = 268.36x + 29.736	0.9767	0.09 ± 0.008
	*Valsa mali*	y = 115.12x + 31.014	0.8673	0.14 ± 0.008

## 3. Experimental

### 3.1. General

ESI-MS was performed on an Agilent 1200 HPLC with the 6130 SQD system, with a Waters XBridge C18 column (50 × 4.6 mm, 3.5 μm, Waters). HR- ESI-MS was performed on Accurate-Mass-Q-TOF-LC/MS 6520 (Agilent, USA). HPLC separation was performed on a Gilson GX-281 HPLC with a PRC-ODS column (20 × 250 mm, 15 μm, Shimadzu). The mobile phase for HPLC was 10 mM NH_4_CO_3_ water solution (A) and MeCN (B) for base method, 0.05% TFA water solution(A) and MeCN (B) for acid method, respectively. ^1^H- and ^13^C- spectra were measured with a Bruker AVANCE III spectrometer (500 MHz) and a Bruker AVANCE III spectrometer (400 MHz). The FTIR spectra was measured with a Shimadzu Prestige-21 spectrometer. Thin layer silica gel was purchased from Qingdao Ocean Company. All solvents were analytical grade.

### 3.2. Plant Material

A 5-year-old whole *Callianthemum taipaicum* plant was collected in Taibai Mountain, Shaanxi Province, China, in September 2010. The voucher specimen (NO. 148) has been deposited at the Herbarium of the Northwest Sci-Tech University of Agriculture and Forestry.

### 3.3. Extraction and Isolation

The air-dried and powdered plant (988 g) of *Callianthemum taipaicum* was extracted five times with 95% ethanol (1 L, 12 h) at room temperature. The filtrates were combined and evaporated to dryness under vacuum. The residue (188 g) was suspended in water (1.8 L) and partitioned with petroleum ether, ethyl acetate and *n*-BuOH, successively. 

The *n*-BuOH extract (14.0 g) was fractionated by column chromatography over silica gel eluting with CH_2_Cl_2_-MeOH (20:1 to 0:100) to yield five fractions (Fr. 1–Fr. 5). Fr. 2 (4.3 g) was submitted to RP-HPLC on PRC-ODS column (20 × 250 mm, 15 μm) with MeCN-H_2_O base method for 0–5 min (5:95) and 5–20 min (50:50) to yield seven subfractions. The subfractions 4–6 were submitted to RP-HPLC on PRC-ODS column (20 × 250 mm, 15 μm) with MeCN-H_2_O (80:20) to yield compound **1** (100 mg), compound **4** (34 mg) and compound **5** (7 mg), respectively. Fr. 3 (2.7 g) was submitted to RP-HPLC on PRC-ODS column (20 × 250 mm, 15 μm) with MeCN-H_2_O base method for 0–5 min (5:95) and 5–20 min (17:83) to yield nine subfractions. Subfraction 5 was submitted to RP-HPLC on PRC-ODS column (20 × 250 mm, 15 μm) with MeCN-H_2_O base method (80:20) to yield compound **6** (14 mg).

The ethyl acetate extract (1.2 g) was submitted to RP-HPLC on PRC-ODS column (20 × 250 mm, 15 μm) with MeCN-H_2_O base method for 0–7.5 min (30:70 to 90:10) to yield five fractions (Fr. 1–Fr. 5). Fr. 2 was submitted to RP-HPLC on PRC-ODS column (20 × 250 mm, 15 μm) with MeCN-H_2_O base method (40:60) to yield compound **2** (18 mg). Fr. 5 was submitted to RP-HPLC on PRC-ODS column (20 × 250 mm, 15 μm) with MeCN-H_2_O acid method (5:95) to yield compound **3** (20 mg).

Compound **1** obtained as a light yellow amorphous powder. Its molecular formula was established as C_27_H_30_O_16_ on the basis of HR-ESI-MS: *m/z* 611.1602 [M+H]^+^ (calcd. [M+H]^+^ 611.1607) and ESI-MS *m/z* 611.0 [M+H]^+^. The IR spectrum showed the characteristic absorption bands of hydroxyl (3444.87 and 3429.43 cm^−1^), carbonyl (1653.00 cm^−1^) and phenyl groups (1600.92 and 1490.97 cm^−1^). ^1^H-NMR (DMSO) and ^13^C-NMR (DMSO): Table 1. 

Compound **2** was obtained as a light yellow amorphous powder. Compound **2** gave D-glucose on acid hydrolysis, identified by comparison with an authentic sample by TLC analysis. ESI-MS: *m/z* 479.0 [M+H]^+^. ^1^H-NMR (DMSO): δ = 12.61 (s, 1H), 10.83 (s, 1H), 9.80 (s, 1H), 7.94 (d, *J* = 2.0 Hz, 1H), 7.49 (dd, *J* = 8.0, 2.0, 1H), 6.91 (d, *J* = 8.0 Hz, 1H), 6.44 (d, *J* = 2.0 Hz, 1H), 6.21 (d, *J* = 2.0 Hz, 1H), 5.57 (d, *J* = 7.2 Hz, 1H), 3.83 (s, 3H), 3.11–3.58 (d, *J* = 11.5 Hz, 2H), 3.11–3.58 ppm (m, 1H); ^13^C-NMR (DMSO): δ = 177.38 (C-4), 164.12 (C-7), 161.20 (C-5), 156.35 (C-9), 156.25 (C-2), 149.37 (C-4'), 146.85 (C-5'), 132.93 (C-3), 122.00 (C-2'), 121.04 (C-1'), 115.18 (C-3'), 113.46 (C-6'), 104.01 (C-10), 100.75 (C-1 of Glc), 98.68 (C-6), 93.67 (C-8), 77.43 (C-5 of Glc), 76.38 (C-2 of Glc), 74.31 (C-4 of Glc), 69.79 (C-3 of Glc), 60.56 (C-6 of Glc), 55.64 ppm (C-21 of OCH_3_). The HMBC spectrum revealed significant correlations of H-6 with C-10; H-8 with C-9 and C-10; H-2' with C-4'; H-3' with C-1' and C-5'; H-5' with C-2' and C-4'; H-1'' with C-3; 5-OH with C-5, C-6 and C-10; 7-OH with C-6, C-7 and C-8; 20-OH with C-3' and C-4'. Based on the above evidence and detailed analysis of the NMR spectra, the structure of compound 2 was determined as isorhamnetin-3-*O*-β-D-glucopyranoside, which had been isolated from *Eupatorium tinifolium* [16].

Compound **3** was obtained as a colorless oily solid. Its molecular formula was established as C_16_H_22_O_4_ by means of ESI-MS *m/z* 279.1 [M+H]^+^. ^1^H-NMR (CDCl_3_): δ = 7.71 (m, 1H), 7.71 (m, 1H), 7.52 (m, 1H), 7.52 (m, 1H), 4.30 (t, *J* = 6.7 Hz, 2H), 1.72 (m, 2H), 1.43 (m, 2H), 0.96 ppm (t, *J* = 7.5 Hz, 3H); ^13^C-NMR (CDCl_3)_: δ = 167.71 (C-7), 132.32 (C-1, C-2), 130.91 (C-4, C-5), 128.84 (C-6), 65.56 (C-8), 30.58 (C-9), 19.18 (C-10), 13.72 ppm (C-11). The HMBC spectrum revealed significant correlations of H-4 with C-2 and C-3; H-5 with C-1 and C-6; H-6 with C-5 and C-7; H-8 with C-7, C-9 and C-10; H-9 with C-8, C-10 and C-11; H-10 with C-8, C-9 and C-11; H-11 with C-9 and C-10, which supported the proposed structure of compound 3. Therefore, the structure of compound **3** was identified as dibutylphthalate, which had been synthesized [17]. 

Compound **4** was obtained as a white amorphous powder. Compound **4** gave D-glucose on acid hydrolysis, identified by comparison with an authentic sample by TLC analysis. ESI-MS: *m/z* 540.2 [M+NH_4_]^+^. ^1^H-NMR (DMSO): δ = 7.05 (d, *J* = 8.5 Hz, 1H), 6.95 (d, *J* = 1.8 Hz, 1H), 6.86 (dd, *J* = 8.6, 1.8 Hz, 1H), 6.74 (d, *J* = 1.2 Hz, 1H), 6.65 (d, *J* = 8.0 Hz, 1H), 6.55 (dd, *J* = 8.0, 1.2 Hz, 1H), 4.86 (d, *J* = 7.6 Hz, 1H), 4.69 (m, 1H), 3.87 (t, *J* = 7.7 Hz, 1H), 3.74 (s, 3H), 3.73 (s, 3H), 3.64 (m, 1H), 3.55 (t, *J* = 7.7 Hz, 1H), 3.47 (m, 1H), 3.10–3.30 (m, 4H), 2.78 (dd, *J* = 13.2, 4.4 Hz, 1H), 2.55 (m, 1H), 2.40 (dd, *J* = 13.2, 11.2 Hz, 1H), 2.18 (m, 1H); ^13^C-NMR (DMSO): δ = 155.06 (C-5'), 148.74 (C-3'), 147.43 (C-3), 145.51 (C-4'), 144.55 (C-4), 137.67 (C-1'), 131.66 (C-1), 120.55 (C-6), 117.73 (C-6'), 115.36 (C-5), 112.70 (C-2), 110.20 (C-2'), 100.16 (C-1 of Glu), 81.60 (C-7'), 76.99 (C-2 of Glu), 76.84 (C-3 of Glu), 73.21 (C-4 of Glu), 71.83 (C-9), 69.68 (C-5 of Glu), 60.66 (C-6 of Glu), 58.58 (C-9'), 55.66 (C-OMe), 55.54 (C-OMe), 52.47 (C-8'), 41.93 (C-8), 32.08 ppm (C-7). The HMBC spectrum revealed significant correlations of H-2 with C-4 and C-6; H-5 with C-1 and C-3; H-6 with C-2 and C-4; H-2' with C-1' and C-3'; H-5' with C-1', C-3' and C-4'; H-6' with C-4'; H-7' with C-9, C-2', C-8' and C-9'; H-1''with C-4'. Based on the above evidence and detailed analysis of the NMR spectra, the structure of compound **4** was identified as (+)-1-hydroxyl pinoresinol-4'-β-D-glucopyranoside, which had been isolated from another plant, *Saussurea japonica* [18]. 

Compound **5** was obtained as a light yellow amorphous powder. Compound **5** gave D-glucose on acid hydrolysis, identified by comparison with an authentic sample by TLC analysis. ESI-MS: *m/z* 538.0 [M+NH_4_]^+^. ^1^H-NMR (DMSO): δ = 8.76 (dd, *J* = 8.2, 1.2Hz, 1H), 8.73 (d, *J* = 8.3 Hz, 1H), 7.05 (d, *J* = 8.5 Hz, 1H), 6.95 (d, *J* = 1.8 Hz), 6.89 (d, *J* = 1.1 Hz, 1H), 4.88 (d, *J* = 3.3 Hz, 1H), 4.67 (d, *J* = 3.6 Hz, 1H), 4.61 (d, *J* = 4.1 Hz, 1H), 4.14 (m, 1H), 3.77 (s, 6H), 3.75 (m, 1H), 3.66 (d, *J* = 11.9 Hz, 1H), 3.45 (dd, *J* = 11.9, 4.5 Hz, 1H), 3.28 (m, 1H), 3.18 (m, 1H), 3.05 ppm (m, 1H); ^13^C-NMR (DMSO): δ= 148.88 (C-3'), 147.48 (C-3), 145.88 (C-4), 145.78 (C-4'), 135.14 (C-1'), 132.11 (C-1), 118.60 (C-6), 118.08 (C-6'), 115.09 (C-5'), 110.45 (C-2'), 110.32 (C-2), 100.06 (C-1 of Glu), 85.13 (C-7), 84.82 ( C-7'), 76.98 (C-5 of Glu), 76.81 (C-3 of Glu), 73.15 (C-2 of Glu), 70.97 (C-9), 69.61 (C-4 of Glu), 60.61 (C-6 of Glu), 55.62 (C-OMe), 55.53 (C-OMe), 53.67 (C-8'), 53.51 ppm (C-8). The HMBC spectrum revealed significant correlations of H-2 with C-1, C-3, C-4, C-6 and C-7; H-7 with C-1, C-2, C-6, C-8 and C-9; H-9 with C-7 and C-8; H-2' with C-1', C-3', C-4', C-6' and C-7'; H-5' with C-1', C-3', C-4' and C-6'; H-6' with C-2', C-4', C-5' and C-7'; H-7' with C-1', C-2', C-6', C-8' and C-9'; H-9' with C-7' and C-8'; H-1'' with C-4'. Thus, the structure of compound **5** was identified as pinoresinol-4'-*O*-β-D-glucopyranoside, which had been isolated from the plant of *Rhus javanica var. roxburghiana* [19].

Compound **6** was obtained as a white amorphous powder. ESI-MS: *m/z* 425.142 [M+Na]^+^. ^1^H- NMR (acetone d_6_): δ = 7.27–7.4 (m, 1H), 4.46 (d, *J* = 7.6 Hz, 1H), 4.46 (d, *J* = 5.7 Hz, 1H), 4.10 (d, *J* = 11.1, 2.3 Hz, 1H), 3.85 (m, 1H), 3.74 (d, *J* = 11.1, 5.9 Hz, 1H), 3.68 (m, 1H), 3.61 (m, 1H), 3.54 (m, 1H), 3.48–3.51 (m, 1H), 3.37–3.43 (m, 1H), 3.27 ppm (m, 1H); ^13^C-NMR (DMSO): δ = 138.4 (C-1), 130.4 (C-2, C-3, C-5, C-6), 130.2 (C-4), 105.3 (C-1''), 103.0 (C-1'), 77.3 (C-3'), 76.6 (C-5'), 74.8 (C-2''), 74.7 (C-2'), 73.2 (C-7), 70.9 (C-4'), 70.3 (C-6'), 66.8 ppm (C-5''). Compound **6** was identified as 2-phenylethyl-β-primeveroside, which had been synthesized [20]. It is reported here as an isolated natural product for the first time.

### 3.4. Acid Hydrolysis of Compound *1*

Compound **1** (5.0 mg) was dissolved in 5.0 mL of 2 N HCl, heated at 90 °C for 2 h, and then partitioned between ethyl acetate and water. Aglycon isorhametin was recovered from the ethyl acetate layer and idertified by direct comparison with an authentic sample. The water layers identified by comparison with an authentic sample by TLC analysis. Sugars liberated from compound **1** were identified as arabinose and glucose [21]. 

### 3.5. Antisepsis Activity

Antifungal activities were assayed using amphotericin as positive control. Four fungi (*Rhizoctonia cerealis*, *Botrytis cinerrea*, *Thanatephorus cucumeris*, *Valsa mali*) were obtained from the Microbe Laboratory of Northwest Sci-Tech University of Agriculture and Forestry, and were identified by Li Xiao-Ming, senior laboratorian. The growth of the hyphae method was adopted to study the compound’s antifungal activity [22]. Every treatment was repeated three times, after cultivation for 72 h at 28.8 °C in constant temperature culture box.

## 4. Conclusions

In this paper, one new flavonol glycoside, one natural product isolated for the first time and other phenolic compounds were isolated from the *Callianthemum* genus. The 2-D NMR data of four known compounds **2–4** were first given. The compounds **1** and **4** exhibited certain antifungal activity. This research provides strong support for further study of *Callianthemum taipaicum*.
